# High Fat-High Fructose Diet Elicits Hypogonadotropism Culminating in Autophagy-Mediated Defective Differentiation of Ovarian Follicles

**DOI:** 10.3390/cells11213447

**Published:** 2022-10-31

**Authors:** Chalikkaran Thilakan Rejani, Ajit Kumar Navin, Thekkey Madathil Valappil Mumthaz, Venugopal Bhuvarahamurthy

**Affiliations:** 1 Department of Medical Biochemistry, Dr. A.L.M. Postgraduate Institute of Basic Medical Sciences, University of Madras, Chennai 600113, Tamil Nadu, India; 2 Department of Pharmaceutical Sciences, Texas A&M Health Science Centre, Texas A&M University, College Station, TX 77843, USA; 3 Department of Zoology, Sir Syed College, Kannur 670142, Kerala, India

**Keywords:** autophagy, GnRHR, hypogonadotropism hypogonadism, folliculogenesis, steroidogenesis, infertility

## Abstract

Pituitary gonadotropins directly govern ovarian functions, which are in turn regulated by the ovarian steroid hormones. The precise interplay of gonadotropins and steroid hormones is critical for follicle growth and differentiation. Furthermore, autophagy regulates ovarian follicle differentiation. However, how the high-fat-high fructose (HFD-HF) diet regulates gonadotropins and facilitates autophagy-mediated follicular differentiation in the ovary is obscure. We fed prepubertal rats (PND 25) an HFD-HF diet until PND 90. The results showed diminished adenohypophyseal GnRHR, PR, and aromatase expression, whereas AR, ERα, PRLR, and inhibin were augmented, resulting in gonadotropins decline. Interestingly, autophagy biomarkers, Beclin-1, ATG5, ATG12, LC3-II, and LAMP1 were reduced but SQSTM1/p62 was augmented in the ovaries of HFD-HF-fed rats, causing autolysosome to aggregation. The diet altered T, E2, P4, PRL, and their receptors status in the ovary, disturbed estrous cyclicity, and delayed vaginal opening. Ovarian histomorphology exhibited numerous cystic and atretic follicles, along with disturbed follicular maturation and ovulation. Moreover, the reduction of FSHR; steroidogenic proteins; receptor proteins AR, ERβ, PR; and signaling proteins Wnt2 and β-catenin was also noticed in the ovary, whereas PRLR, inhibin, and pGSK3β were augmented. In conclusion, exposure to a prepubertal HFD-HF diet leads to hypogonadotropism and the autophagy-mediated defective differentiation of ovarian follicles, abating fertility in adult rats.

## 1. Introduction

Nutritional status determines the attainment of reproductive potential in an individual. Children consume palatable calorie-dense foods during the key phase of development. The consumption of junk food during travel time, restaurants, home, and school in school-going adolescents contributes to poor growth outcomes [1]. National Health and Nutrition Examination Surveys (2003–2004, 2005–2006) report that the highest sources of energy for 2- to 18-year-olds were grain desserts, pizza, and soda, which are low in beneficial nutrients but high in solid fats and/or added sugars [2]. As per recent data, the consumption of saturated fat and sugar in children continues to exceed the recommended limit of fewer than 10% of total calories for anyone 2 years old or older, as boys and girls (age 1–18) obtain a range of about 11–12% of their total calories on average from saturated fat and a range of about 11–17% of their total calories on average from added sugar [3]. In developed countries, and developing countries as well, people highly rely on Western pattern diets, which include proteins (derived from fatty domesticated and processed meats), saturated fats, refined grains, sugar, alcohol, salt, and corn-derived fructose syrup with lower consumption of fruits and vegetables [4]. Moreover, fructose is utilized commercially as a sweetening alternative (fructose corn syrup) for glucose or sucrose in desserts, sauces, and carbonated beverages [5]. These unhealthy fast foods, convenience foods, energy-dense snacks, and soft drinks have resulted in serious overweight, obesity problems, diabetes, and various reproductive problems, such as PCOS, infertility, etc. [6].

The gonadotropes in the adenohypophysis secrete gonadotropins (FSH and LH), which control ovarian functions, such as folliculogenesis and steroidogenesis, and are essential for follicle development, specifically beyond the secondary stage and oocyte maturation [7,8]. LH and FSH are regulated by the hypothalamic gonadotropin-releasing hormone (GnRH) through its own receptor GnRHR. The steroid hormones (T, E2, and progesterone (P4)) act as a connecting link to the hypothalamus-pituitary–ovary (HPO) axis and tightly regulate it by maintaining a proper set-point through a feedback mechanism. This complex regulation might have a detrimental effect when pathologies occur within any juncture of the HPO axis [8]. In the ovary, the granulosa cells (GCs) are vital to the formation of follicles and fate determination during folliculogenesis [9,10]. GCs proliferate and differentiate in tandem with follicular growth, which is crucial for oocyte development and female fertility [11], albeit with the involvement of gonadotropins and several transcription factors, such as GATA4, GATA6, FOXO1, etc. [12,13,14]. Ovarian follicles can either produce oocytes or undergo follicular atresia. Moreover, the HPO endocrine system, the Wnt2/GSK3β/β-catenin signaling pathway [15], and autophagy cellular processes [16] regulate follicular development in the ovary. The process of autophagy involves the maturation and fusion of autophagosomes with lysosomes for intracellular degradation. Autophagy is governed by various autophagy related-genes, such as BECL1 (involved in the formation of pre-autophagosomes), LC3-II (Microtubule-associated protein1 light chain 3, a marker of autophagosome), ATG5 (a member of Atg5-Atg12–Atg16L complex involves with the extending phagophore), p62/SQSTM1 (a multi-functional adaptor molecule promotes turnover of polyubiquitinated protein aggregates), etc. [17]. The improper regulation or mutations of these genes highly influence follicular development and thereby female reproduction. In the ovary, autophagy serves as a cell death mechanism in atretic follicles and degenerating corpora lutea [18]. Autophagy contributes to the apoptosis of GCs to accelerate follicular atresia [19]. The lack of Becl1 in ovarian GCs showed defects in P4 production subsequently resulting in preterm labor [20].

Various studies reported that dietary imbalances disrupt the development of reproductive processes [21]. Clarke et al. (1997) explored whether an increase in utero estrogen exposure, as produced by the consumption of HFD during pregnancy, could alter mammary gland development, puberty onset, and breast cancer-susceptibility in offspring. Mirzaei et al. (2022) reported that increased FSH and LH levels, decreased serum T and E2 levels, and strong AR expression in the ovaries of adult rats fed with high fructose corn syrup [22]. Ruxton et al. (2010) delineated that the consumption of high amounts of refined carbohydrates in food and beverage increases the risk of dyslipidemia, obesity, insulin resistance, heart disease, and female reproductive issues, such as endometriosis, dysmenorrhea as well [22,23]. Exposure to HFD elevated the levels of serum LH and P4 prolonged the estrous cycle and altered ovarian morphology in rats [24]. Another study also ascertained the appearance of metabolic disturbances and ovarian changes that resembles patients with polycystic ovarian syndrome (PCOS) in the HFD-fed rat from a pre-pubertal age [25]. Hussain et al. (2016) reported impaired estrous cyclicity, precluded conception, and high offspring mortality in female rats fed with a high-fat-high-sugar diet [26].

Evidence accrued from various studies unveils the impairment of reproductive functions due to a diet enriched with high fat and high fructose (sugar). However, the regulation of gonadotropins in the anterior pituitary and the detrimental effect on ovarian functions, particularly autophagy-mediated follicles growth and differentiation in the HFD-HF-fed adult rats, are obscure. Thus, we hypothesized that “pre-pubertal exposure to HFD-HF diet may interfere with the specific proteins that regulate pituitary gonadotropins and key autophagy biomarkers leading to defective ovarian follicular growth and differentiation in adult rats.” In this investigation, we show that HFD-HF diet reduced GnRHR on pituitary gonadotropes, which resulted in decreased gonadotropins level as well as a disruption of a hormonal interplay of gonadotropins regulation. The study also reveals that the HFD-HF diet disrupts the Wnt2/GSK3β/β-catenin signaling pathway and thereby follicle growth, while the inhibition of autophagy leads to the defective differentiation of ovarian follicles.

## 2. Materials and Methods

### 2.1. Chemicals and Reagents

Acrylamide, bis-acrylamide, ammonium persulfate, and TEMED were purchased from Bio-Rad, CA, USA. The β-actin (Sigma-Aldrich, St. Louis, MO, USA) antibody, Bovine Serum Albumin (BSA), was purchased from Sigma Aldrich Chemicals Pvt. Ltd., St. Louis, USA. Polyvinylidene difluoride (PVDF) membrane was purchased from Millipore, Bangalore, IN. Primary antibodies ERα (SC-542), ERβ (SC-8974), AR (SC-815), GnRHR (SC-8682), PR (SC-398898), PRLR (SC-20992), wnt-2 (SC-514382), β-catenin (SC-797), Inhibin (SC-6306), CYP17A1(SC-46080), CYP11A1 (SC-18040), CYP19 (SC-14245), FSHR (SC-13935), LHR (SC-25828), StAR (SC-23523), 3βHSD (SC-30820), and 17βHSD (SC-26968) were purchased from Santacruz Biotechnology (TX, USA). ATG5 (CST 12994S), ATG12 (CST 4180S), Beclin-1 (CST 3495S), LC3-II (CST 12741S), SQSMT/p62 (95697S), GSK-3β (CST 9315), and pGSK-3β (CST 9336), were purchased from Cell Signaling Technologies, MA, USA. LAMP-1(Ab 25630) was purchased from Abcam, MA, USA. Secondary antibodies, HRP, and Biotin conjugated anti-mouse IgG, anti-rabbit IgG, and anti-goat IgG were obtained from Genei, Bangalore, India. Methanol, Cholesterol, Cholic acid, Fructose, and all other chemicals were purchased from SRL, New Delhi, India, and were of analytical grade. ECL kit (Pierce ECL Western Blotting Substrate) was obtained from Thermo Scientific, IL, USA.

### 2.2. Animals

The experiments performed in nulliparous adult female Sprague Dawley rats (*Rattus norvegicus*) were approved by the Institutional Animal Ethics Committee for studies on experimental animals (Ref: IAEC No: 02/07/2021), which had a nominee of the Committee for Prevention of Cruelty and Safety to Experimental Animals (CPCSEA), Government of India, New Delhi. All rats were maintained in clean polypropylene cages at the Central Animal House Facility, Dr. ALM PG IBMS, University of Madras, Taramani Campus, Chennai-600113 (T.N.), India.

### 2.3. Diet Composition and Treatment Period

Pre-pubertal rats (PND 25) were divided into 2 groups: Group I (Control), where rats were fed a standard pelleted diet (Lipton, India) which contained 3% fat, 21% protein, and 48% carbohydrate as a major content and provided regular drinking water ad libitum, and Group II (HFD-HF diet exposed rats), where the experimental rats were fed on an HFD-HF diet. The HFD-HF diet composition consists of cholesterol (3%), cholic acid (1%), and coconut oil (30%) along with standard rat feed (66%) as well as fructose (25%) mixed in drinking water. The experimental rats, after weaning (PND 25), were provided an HFD-HF diet until PND 90, which covered the pre-pubertal, pubertal, and adult periods. The diet was provided after weaning to examine the detrimental reproductive impairments through the natural progression of development. The rats were weighed every other day throughout the study. The rats were kept in a climate-controlled setting with a 12:12 h light/dark cycle. At the end of 90 days, rats were anesthetized in the diestrous phase by sodium thiopentone (40 mg/kg body weight) (i.p.). The blood was collected to obtain serum for the assessment of hormones. The tissue samples (pituitaries and ovaries) were collected, weighed individually, and calculated for the respective relative weights (=Organ weight/Body weight) before evaluating the expression of proteins and other necessary parameters.

### 2.4. Hormone Analysis by ELISA Method

Serum was collected from blood and assessed for hormonal status. An enzyme-linked immunosorbent assay (ELISA) was performed by using a commercial kit to measure serum hormone levels of FSH and LH (Immunotag ELISA kits, Geno Technology Inc., USA), testosterone, estradiol, prolactin, and progesterone (Cal biotech ELISA kits, CA). The sensitivity of FSH and LH were <1.406 mlU/mL; testosterone, progesterone, prolactin were <0.2 ng/mL; and estradiol was <0.2 pg/mL, respectively.

### 2.5. Western Blot Analysis

Ovarian tissues (left ovary) were washed with ice-cold PBS and added to 500 µL of ice-cold RIPA buffer (prepared by using 50 mM Tris, 150 mM NaCl, 1 mM EDTA, 0.5% Sodium deoxycholate, 0.1% SDS, 1% Triton-X100) with protease and phosphatase inhibitors. The tissues were homogenized, centrifuged at 12,000 rpm at 4 °C for 15 min, supernatant was collected, and protein concentration using the Bradford method was determined. Of the total protein, 50 µg was mixed with 2 X sample buffer, heated for 5 min, and 10% SDS-PAGE electrophoresis was performed. Following electro-transfer (wet method) on a PVDF membrane (Millipore, Germany) it was blocked in the buffer containing 5% non-fat dry milk for 2–3 h, washed, and incubated overnight after adding primary antibody (1:1000 dilution). Membranes were further incubated with respective horseradish peroxidase-conjugated secondary antibodies (1:5000 dilutions) at room temperature for 1 h, and the signals were detected and visualized using ECL (Enhanced chemiluminescence) reagent in Chemidoc XRS (Bio-Rad, USA). Band intensity was quantified by Quantity One 1-D Analysis Software 4.6.6 (Bio-Rad, CA, USA). β-actin was used as an internal control.

### 2.6. Histology and Follicular Assessment of Ovaries

The ovaries of experimental rats were removed and fixed in 4% paraformaldehyde (PFA) (Fisher Scientific, Mumbai, India). For processing, the right ovary was hydrated, dehydrated in the series of alcohol, embedded in paraffin wax at 58 °C, and sectioned at 5 µm. Tissue sections were stained with Hematoxylin and Eosin (H&E). Photos were captured with the Nikon Eclipse 80i microscope at various magnifications (Nikon Instruments, Tokyo, Japan). H&E-stained slides were evaluated by light microscopy and changes in histoarchitecture were recorded. Every 10^th^ serial section per ovary was assessed for ovarian follicular counts, differentiation, and degeneration. For each rat, the total number of follicular cysts, corpora lutea (CL), and follicle types were estimated, as per the criteria previously described [9,27]. Primordial follicles were identified when the ovum was encircled by loosely bound GCs. In primary follicles, the ovum was surrounded by a single layer of GCs, while in secondary follicles by double layers of GCs. The antral follicles were identified with a distinct antral cavity; a thick and homogenous layer of GCs; and a thin outline of theca cells. CLs were detected based on tissue density (containing at least >60% area) and the presence of luteinized cells with a granular appearance. A clear antral cavity; a thick, homogeneous layer of GCs; and a thin, defined contour of theca cells were used to identify mature follicles. Moreover, the ovum was also firmly attached to the GCs layer in an oval-shaped follicle. Follicular cysts were identified as follicles without an ovum that had a wide antral gap surrounded by an expanded and highly stained theca cell layer. To determine the number of follicular structures per section, the total number of follicular structures per ovarian tissue segment was divided by the number of sections for that ovary.

### 2.7. Immunohistochemistry

The rats were anesthetized with sodium thiopentone (40 mg/kg body weight) (i.p.) and perfused with 0.9% NaCl solution injected into the left ventricle 20 min/mL of 200–300 gm body weight. The right atrium was opened to facilitate the passage of injected saline. Thus, each experimental animal was transcardially perfused, and pituitaries were fixed in 4% PFA. For processing, pituitaries were hydrated, dehydrated in the series of alcohol, blocked in paraffin wax at 58 °C, and sectioned at 5 µm. The pituitary sections were processed for immunohistochemistry by dewaxing in xylene followed by a series of alcohol washes. The sections were immersed in citrate buffer (pH 6.0) and heated at 95 °C for 5 min for antigen retrieval. The blocking of endogenous peroxidase activity was performed by treating the sections with 3% H_2_O_2_ in methanol for 20 min. The blocking of nonspecific protein interactions was then performed using 1% bovine serum albumin (BSA) for 1 h followed by overnight primary antibodies incubation at 4 °C. The tissue sections were further incubated with the secondary antibody (anti-rabbit IgG or anti-goat IgG) (1:300 μL) for 1 h at room temperature, introduced with avidin/biotin conjugated to peroxidase (Vectastain ABC detection systems, Vector Laboratories, Inc. USA), exposed to 3, 3′ diaminobenzidine tetrahydrochloride (DAB) (Impact, Vector Labs, Burlingame, CA), and counterstained with hematoxylin. After mounting in DPX (Dibutyl phthalate polystyrene Xylene), the stained sections were observed under a Nikon Eclipse 80i microscope (Japan), and photographs were taken using NIS element software at 400 X magnification. The intensity of staining for each protein was quantified using Image-Pro Plus 10.6 image processing and analysis software according to the manufacturer’s instructions (Media Cybernetics, Inc., Bethesda, MD). Briefly, six images of 400 X magnification were captured randomly without hot-spot bias in each tissue section per animal. The integrated optical density (IOD) of immunostaining was quantified under RGB mode. Numerical data were expressed as mean ± SEM.

### 2.8. RNA Extraction and Real-Time Reverse Transcriptase Polymerase Chain Reaction (RT-PCR) Analysis

Messenger RNA was extracted from the ovary using RNAeasy Mini Kit (Qiagen, Hilden, Germany) as per the manufacturer’s instruction. The purity (1.8–2.0) and concentration of RNA were determined spectrophotometrically at a 260/280 nm absorbance for real-time RT-PCR analysis. Furthermore, cDNA synthesis by reverse transcription using iScript DNA Synthesis Kit (Bio-Rad, Hercules, CA) was performed, and RT-PCR was conducted using SYBR Green PCR Master Mix (Applied Biosystems, Foster City, CA) and sense and antisense oligonucleotide primers for the respective genes with specific primers (Table 1: specific primer sequence). GAPDH was used as an internal control. The reaction cycles were as follows: PCR enzyme initial activation at 95 °C for 15 min, initial denaturation at 94 °C for 15s, annealing at 56 °C for 30s, and elongation at 72 °C for 30s for 40 cycles. All reactions were run in triplicate. The PCR amplification of all transcripts was performed on a CFX96 Touch Real-Time PCR machine (Bio-Rad, CA, USA). The fold differences were calculated by normalizing the relative expression of the gene of interest with GAPDH and the results were expressed as fold changes.

### 2.9. Statistical Analysis

For comparisons between groups (Control and HFD-HF diet-exposed group), all results are expressed as the mean +/− SEM. The numerical variables between the 2 groups were compared using an unpaired Student’s *t*-test. All statistical analyses were conducted using GraphPad Prism, version 8.0 (GraphPad, La Jolla, CA, USA). Differences were considered significant with *p ≤* 0.05 values.

## 3. Results

### 3.1. Body Weight, Pituitary Weight, Ovary Weight, Vaginal Opening, and Cyclicity

**Figure 1:**
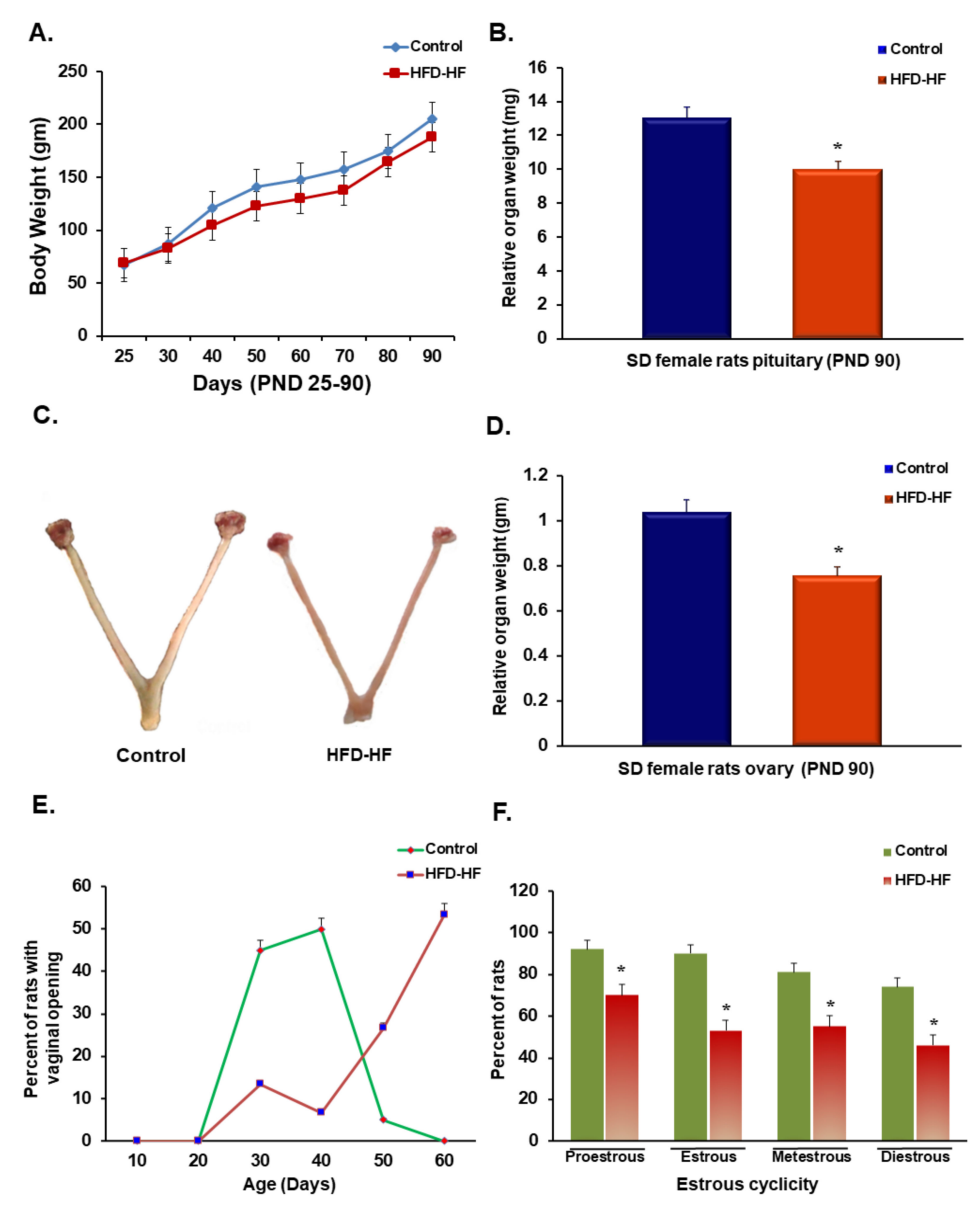
Effect of prepubertal exposure to HFD-HF diet on (**A**) body weight, (**B**) relative pituitary weight, (**D**) relative ovary weight of adult rats, (**E**) vaginal opening, and (**F**) estrous cyclicity. A representative picture (**C**) shows the ovary of the control and HFD-HF-fed rat. Each value is the mean ± SEM of six animals in each group. Bars with an asterisk (*) denote statistically significant differences between such means at *p ≤* 0.05 level.

Rats subjected to the HFD-HF diet during the pre-pubertal period showed unaltered body weight, compared to control adult female rats (Figure 1A,C). Interestingly, the relative weight of the pituitary and ovary were significantly reduced in the rats exposed to the HFD-HF diet (Figure 1B,D). The vaginal opening is the marker of the onset of puberty [23]. Estrous cyclicity was negatively affected in rats fed on the HFD-HF diet during the prepubertal age (Figure 1F). The data showed that >90% of control rats achieved normal cycling activity (lasting for 4–5 days) after vaginal opening on 35–40 days normally, whereas a delayed vaginal opening (visible only after 50–60 days) was observed (Figure 1E) in the rats exposed to HFD-HF diet with less than 30% rats only reached to normal cycling.

### 3.2. Serum Hormone Profiles

**Figure 2:**
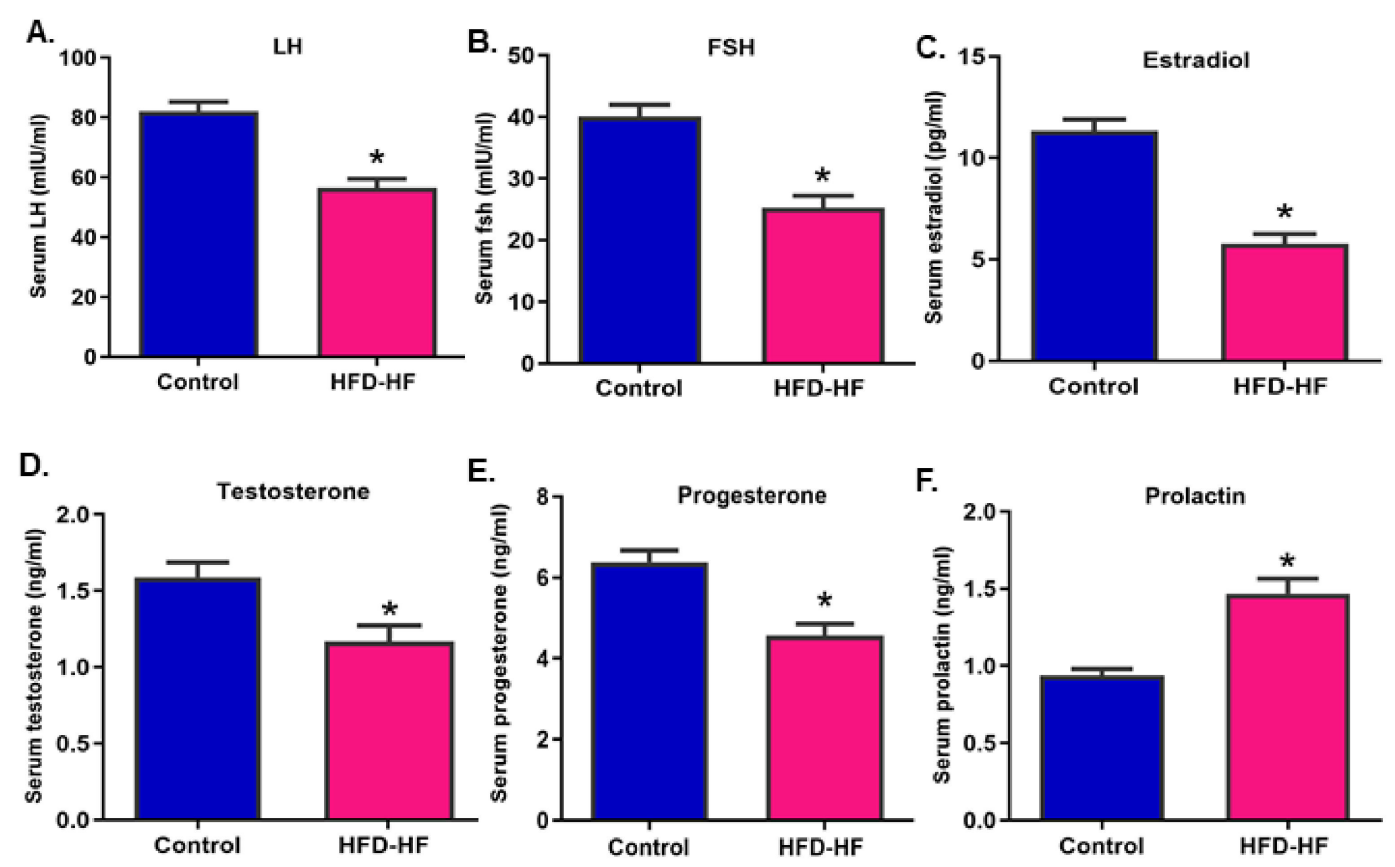
Serum titers of (**A**) luteinizing hormone, (**B**) follicle-stimulating hormone, (**C**) estradiol, (**D**) testosterone (**E**) progesterone, and (**F**) prolactin in adult rats subjected to an HFD-HF diet. Each value is mean ± SEM of six animals, in each group. Bars with the asterisk (*) sign denote statistically significant differences (*p ≤* 0.05 vs. control group) between the respective means.

The serum levels of LH, FSH, E2, T, and P4 were decreased (Figure 2A–E), whereas the prolactin (Figure 2F) level increased significantly (*p* ≤ 0.05) in the rats subjected to the HFD-HF diet, compared to control.

### 3.3. Histoarchitecture of the Ovary and Follicular Assessment

**Figure 3:**
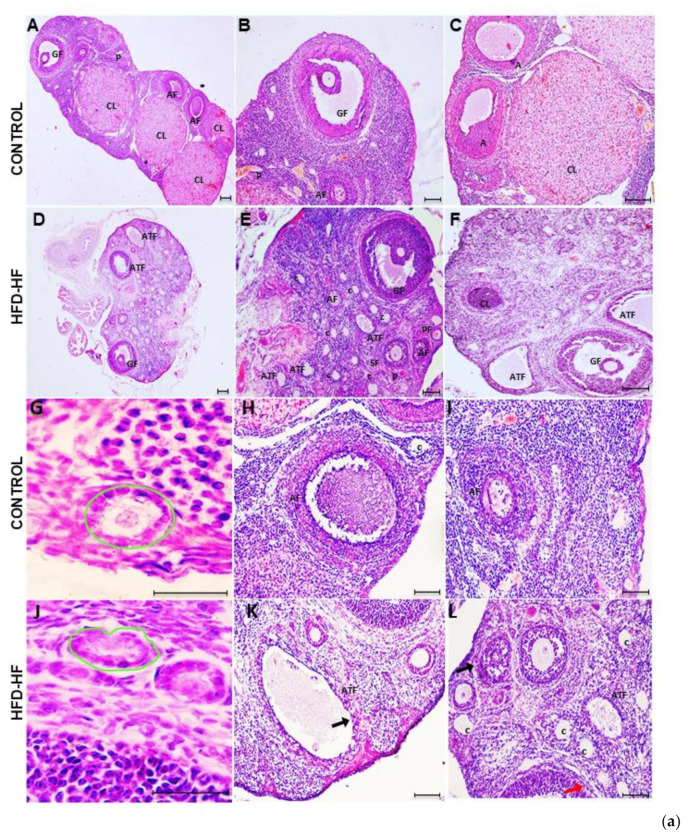

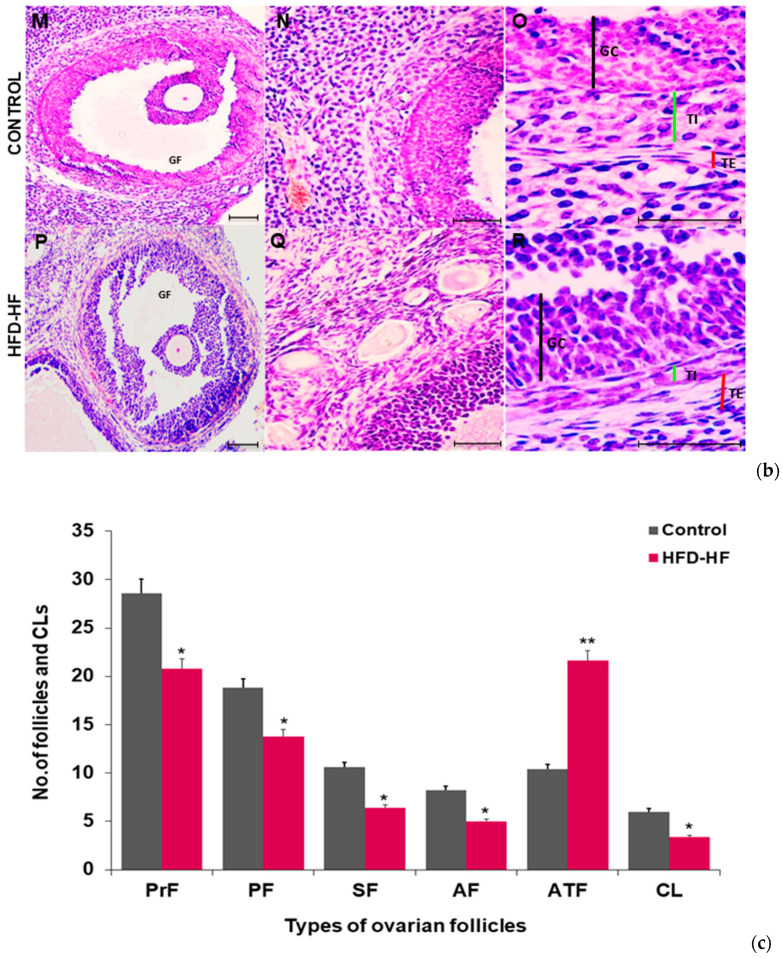
The representative photomicrographs of the ovary of control adult rats exhibiting normal histoarchitecture with clear primordial follicles, primary follicles, secondary follicles, antral follicles, and preovulatory and ovulatory follicles, along with corpus luteum ((**a**)**A**–**J**). A compact arrangement of granulosa cells (GCs) and theca cells (TCs) with a clear demarcation line in the ovary of control rats ((**a**)**A**–**C**,**G**–**I**;**(b)M**–**O**) is visible, whereas the ovary of the adult rats subjected to HFD-HF diet showing disturbed histoarchitecture with a thin layer of TCs and disintegrated GCs, a few fresh corpora lutea, a large number of follicular cysts and atretic follicles ((**a**)**D**–**F**,**J**–**L**; (**b**)**P**–**R**). (Scale bar = 100 μm) (**c**) A histogram represents the numbers of counted follicles and corpora lutea in the ovary of rats fed on the HFD-HF diet, compared to the control. Abbreviation: CL: corpora lutea; TC: thecal cells, GC: granulosa cells, C: follicular cysts; TI; Theca interna layer; TE: Theca externa, PrF: primordial follicles, PF: primary follicle, SF: secondary follicles, AF: antral follicles, ATF: atretic follicle. Each bar is the mean ± SEM of three observations from six animals of each group. The (*, **) signs noted on the bars represent statistically significant differences between the respective means (* *p* ≤ 0.05, ** *p* ≤ 0.01 vs. control group).

The ovary of the control rat showed normal histomorphology containing various types of follicles in different stages, i.e., primordial follicles, primary follicles, secondary follicles, and pre-antral and antral follicles. Graafian follicles were well-developed with oocytes surrounded by cumulus oophorous, and corpora lutea were also evident throughout the section [Figure 3(aA–C)]. H&E staining exhibited altered ovarian morphology in adult rats exposed to the HFD-HF diet. The ovary of HFD-HF-fed rats (PND 90) displayed morphological alterations, including a considerable number of atretic follicles [Figure 3(aE,F,L)], follicular cysts [Figure 3(aK)], disintegrated Graafian follicles, and fewer corpora lutea [Figure 3(aE,F)], compared to control [Figure 3(aA,C)]. Moreover, the ovary of HFD-HF-fed rats exhibited constricted primary follicles, atretic follicles with thinning of GCs layers as well as perturbed secondary follicles [Figure 3(aJ–L)] compared to the corresponding control [Figure 3(aG–I)]. In addition, the layers of GCs, theca interna, and theca externa surrounding the oocyte in the ovary of the control rat appeared to have normal compaction [Figure 3(bM–O)], whereas these layers were disorganized with loosely arranged and expanded GCs width, reduced theca interstitial cell layer, and disturbed theca externa in the ovary of rats fed on the HFD-HF diet [Figure 3(bP–R)]. The number of primordial follicles, primary and secondary follicles, antral follicles, atretic follicles, and corpora lutea were counted in the ovary of the control and HFD-HF-fed rats and expressed as mean+/− SEM [Figure 3c].

### 3.4. Immunohistochemical Localization of Gonadotropin Regulatory Proteins in the Pituitary

**Figure 4:**
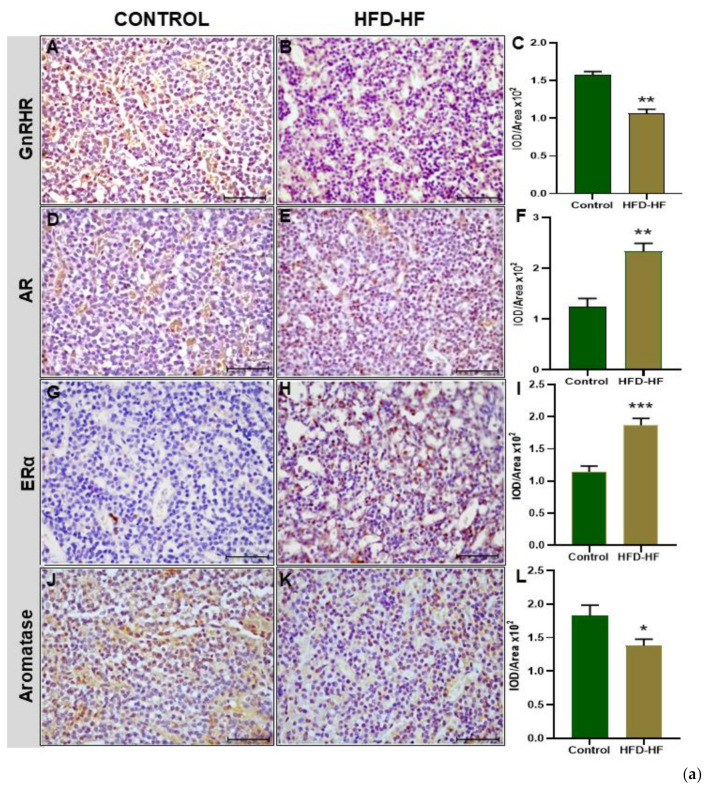

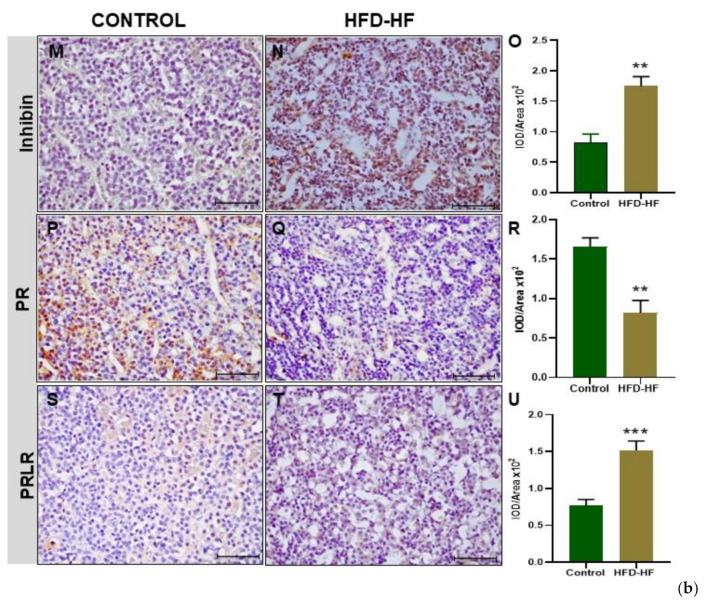
Immunohistochemical localization of gonadotropins and regulatory proteins in the anterior pituitary. (**a**) Immunohistochemical localization of GnRHR (**A**–**C**), AR (**D**–**F**), ERα (**G**–**I**), and P450 aromatase (**J**–**L**) protein in the pituitary of adult rats fed on HFD-HF diet (**A**–**J**) and control rats (**B**–**K**). (**b**) Immunohistochemical localization of Inhibin (**M**–**O**), progesterone receptor (PR) (**P**–**R**), and prolactin receptor (PRLR) (**S**–**U**) protein in the pituitary of adult rats subjected to the HFD-HF diet and control rats. Each bar is the mean ± SEM of the observations from six animals from each group. The (*, **, ***) signs noted on the bars represent statistically significant differences (* *p* ≤ 0.05, ** *p* ≤ 0.01, *** *p* ≤ 0.0001 vs. control group) between the respective means. [Scale bar = 100 μm].

Prepubertal exposure to the HFD-HF diet significantly decreased (*p* ≤ 0.05) the immunopositivity of GnRHR in the adenohypophysis of adult rats, compared to the control (Figure 4A–C). The immunostaining of AR protein in the adenohypophysis of the rats fed on the HFD-HF diet exhibited increased expression (*p* ≤ 0.05), compared to the control (Figure 4D–F). The HFD-HF diet increased the immunostaining of ERα protein in the anterior pituitary as well, compared to the control rats (Figure 4G–I), whereas P450 aromatase showed a significant reduction (*p* ≤ 0.05) (Figure 4J–L). An intense immunopositivity of inhibin β was observed in the anterior pituitary cells of the HFD-HF diet-fed rats as compared to control rats (Figure 4M–O). A decreased expression of progesterone receptor (PR) was observed in rats fed on the HFD-HF diet, compared to the control (Figure 4P–R). The prolactin receptor (PRLR) in the anterior pituitary of the rats subjected to the HFD-HF diet exhibited increased intensity (*p* ≤ 0.05), compared to the control (Figure 4S–U).

### 3.5. Expression of Gonadotropin Receptors and Steroidogenic Enzymes in the Ovary

**Figure 5:**
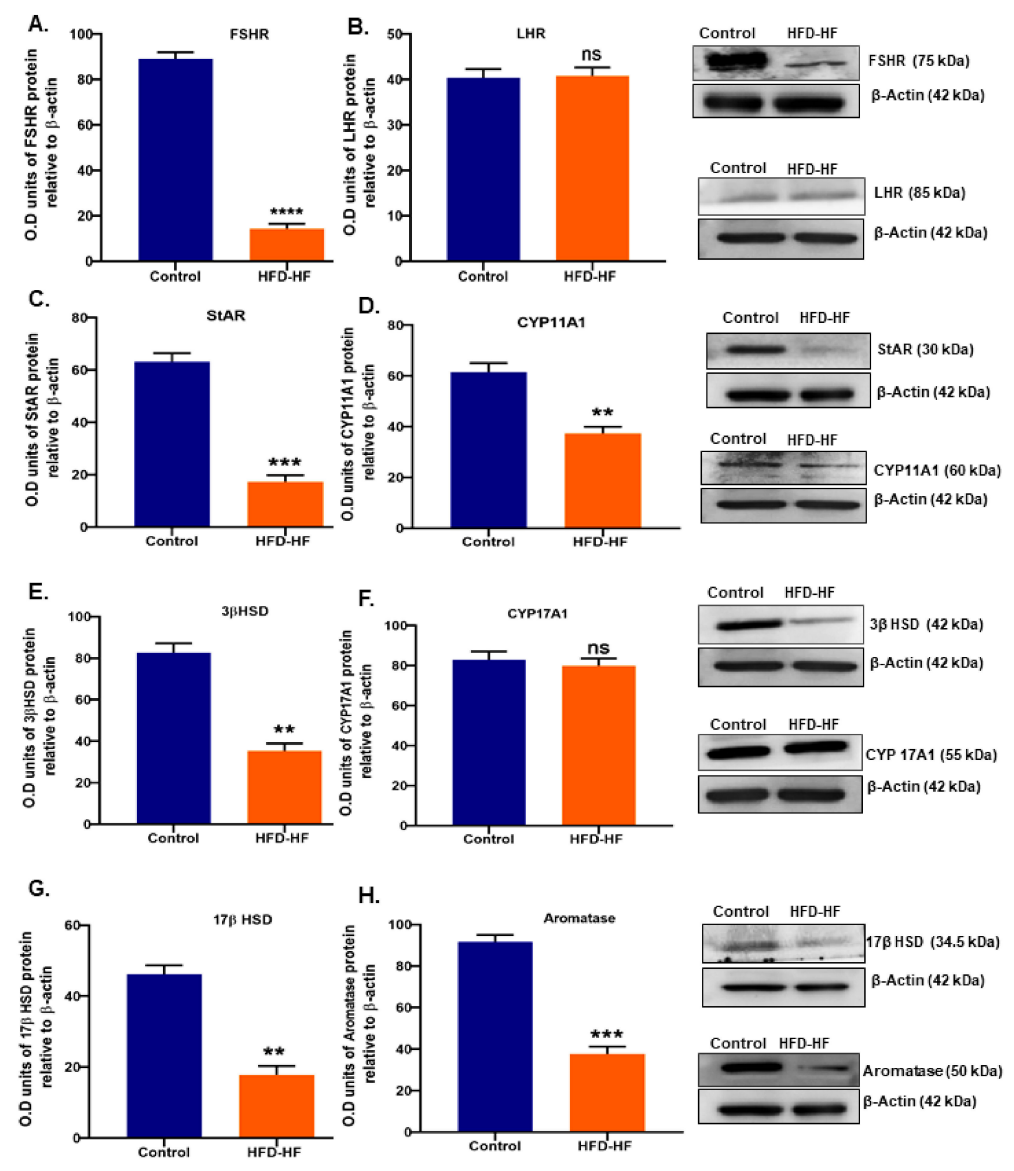
Effects of the HFD-HF diet on the expression level of (**A**) FSHR, (**B**) LHR, (**C**) StAR, (**D**) CYP11A1, (**E**) 3βHSD, (**F**) CYP17A1, (**G**) 17βHSD, and (**H**) P450 aromatase in the ovaries of rats fed on the HFD-HF diet. Each value is mean ± SEM of three samples of pooled cells from six animals from each group. Bars with ns denote statistically insignificant differences between the respective means, while those (**, ***, ****) signs represent statistically significant differences (** *p* ≤ 0.01, *** *p* ≤ 0.0001, **** *p* ≤ 0.0001 vs. control group) between the respective means.

Western blot detected the decreased expression of FSHR (Figure 5A), whereas LHR remained unchanged (Figure 5B) in the rats exposed to the HFD-HF diet, compared to the control. The expression of key steroidogenic proteins in the ovary of HFD-HF diet-fed rats showed decreased StAR, CYP11A1, 3βHSD, and 17βHSD (Figure 5C–E,G) as well as testosterone metabolizing enzyme P450 aromatase (Figure 5G), although CYP17A1 expression unaltered (Figure 5F).

### 3.6. Expression of Ovarian Receptors Proteins and Inhibin Protein

**Figure 6:**
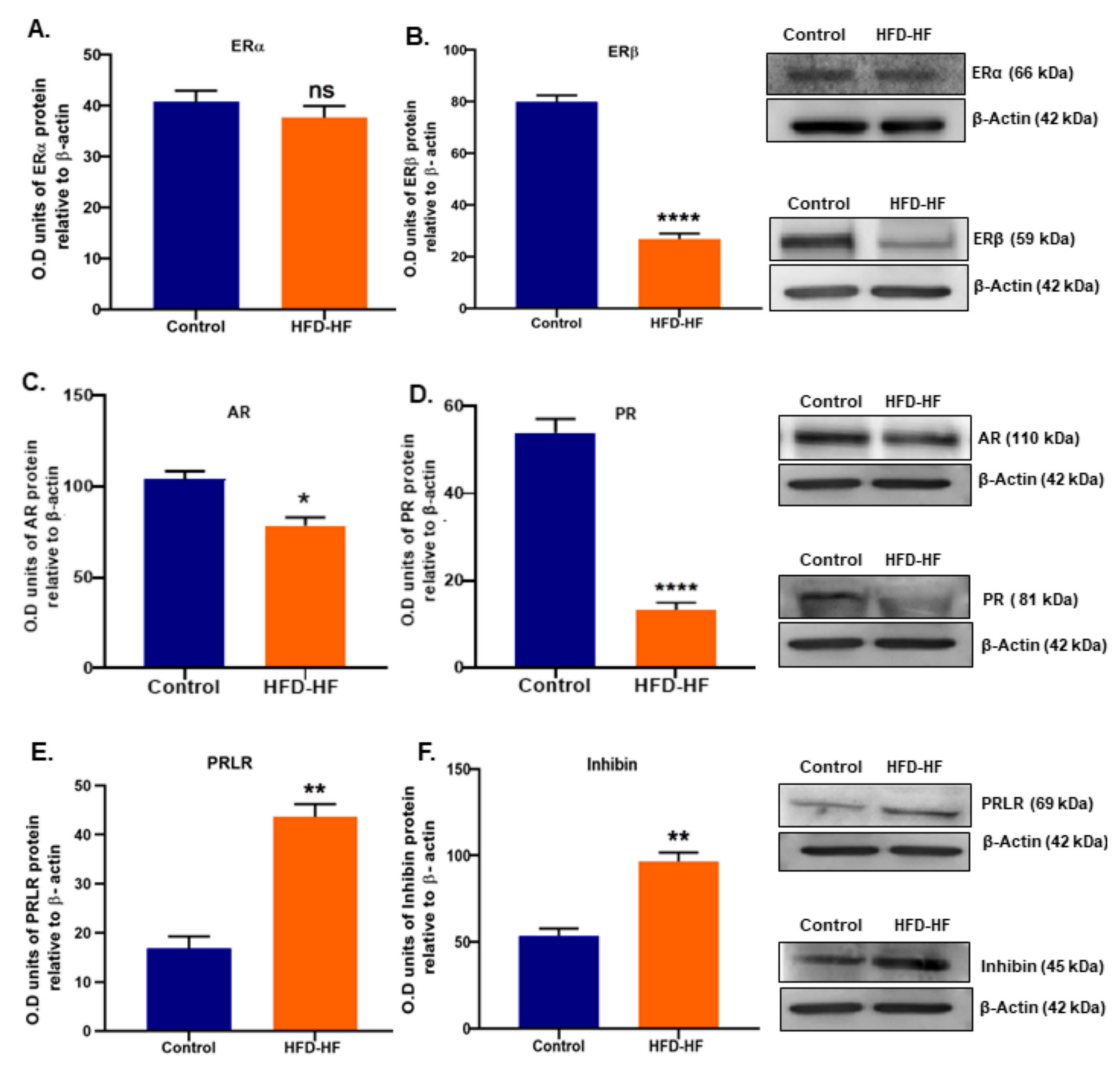
Effects of the HFD-HF diet on the expression level of (**A**) ERα, (**B**) ERβ, (**C**) AR, (**D**) PR, (**E**) PRLR, and (**F**) Inhibin in the ovary of the adult rat. Each value is mean ± SEM of three samples of pooled cells from six animals in each group. Bars with ns denote the statistically insignificant differences between the respective means, while those (*, **, ****) signs represent statistically significant differences (* *p* ≤ 0.05, ** *p* ≤ 0.01, **** *p* ≤ 0.0001 vs. control group) between the respective means.

Pre-pubertal exposure to HFD-HF diets decreased ERβ, AR, and PR proteins (Figure 6B–D), whereas PRLR increased (Figure 6E) in the ovaries of rats. Interestingly, the level of ERα in the adult rats did not show any significant difference between HFD-HF diet-exposed rats and the control (Figure 6A). Inhibin, an FSH regulator, increased in the ovary of adult rats fed on the HFD-HF diet, compared to the control (Figure 6F).

### 3.7. Effect of HFD-HF on Proteins of the Ovarian Wnt2/GSK3β/β-Catenin Pathway

**Figure 7:**
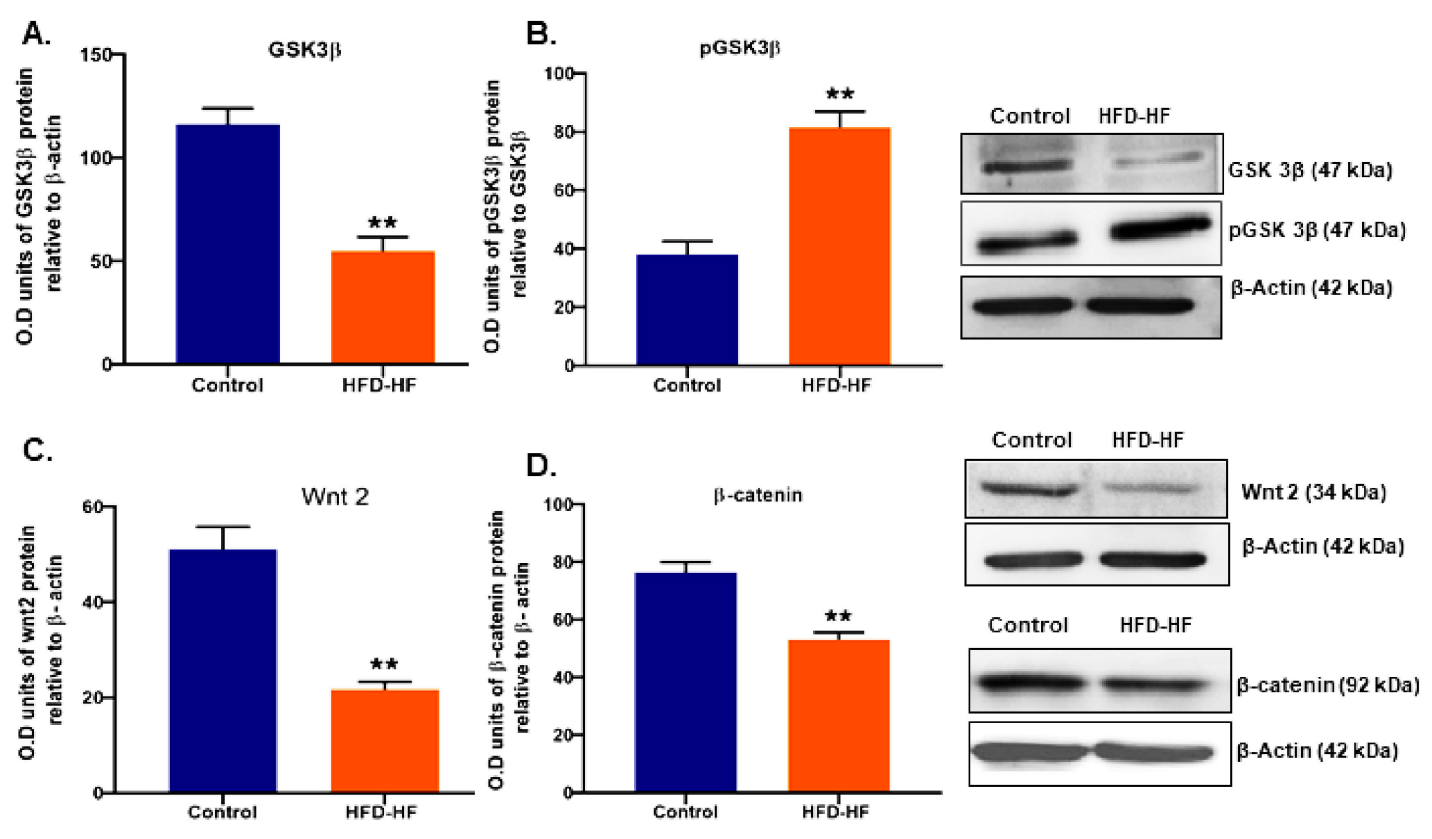
Effects of the HFD-HF diet on the expression level of (**A**) GSK3β, (**B**) pGSK3β, (**C**) Wnt2, and (**D**) β-catenin in the ovaries of adult rats. Each value is mean ± SEM of the three samples of pooled cells from six animals from each group. Bars with (**) signs represent statistically significant differences (** *p* ≤ 0.01 vs. control) group between the respective means.

The key regulatory proteins Wnt2, GSK3β, and β-catenin of the Wnt2/GSK3β/β-catenin signaling pathway involved in ovarian folliculogenesis showed significantly decreased (*p* ≤ 0.05) expression (Figure 7A–D), whereas the expression of pGSK3β protein increased (Figure 7B).

### 3.8. Effect of HFD-HF Diet on the Expression of Key Autophagy Genes and Proteins in the Ovary

**Figure 8:**
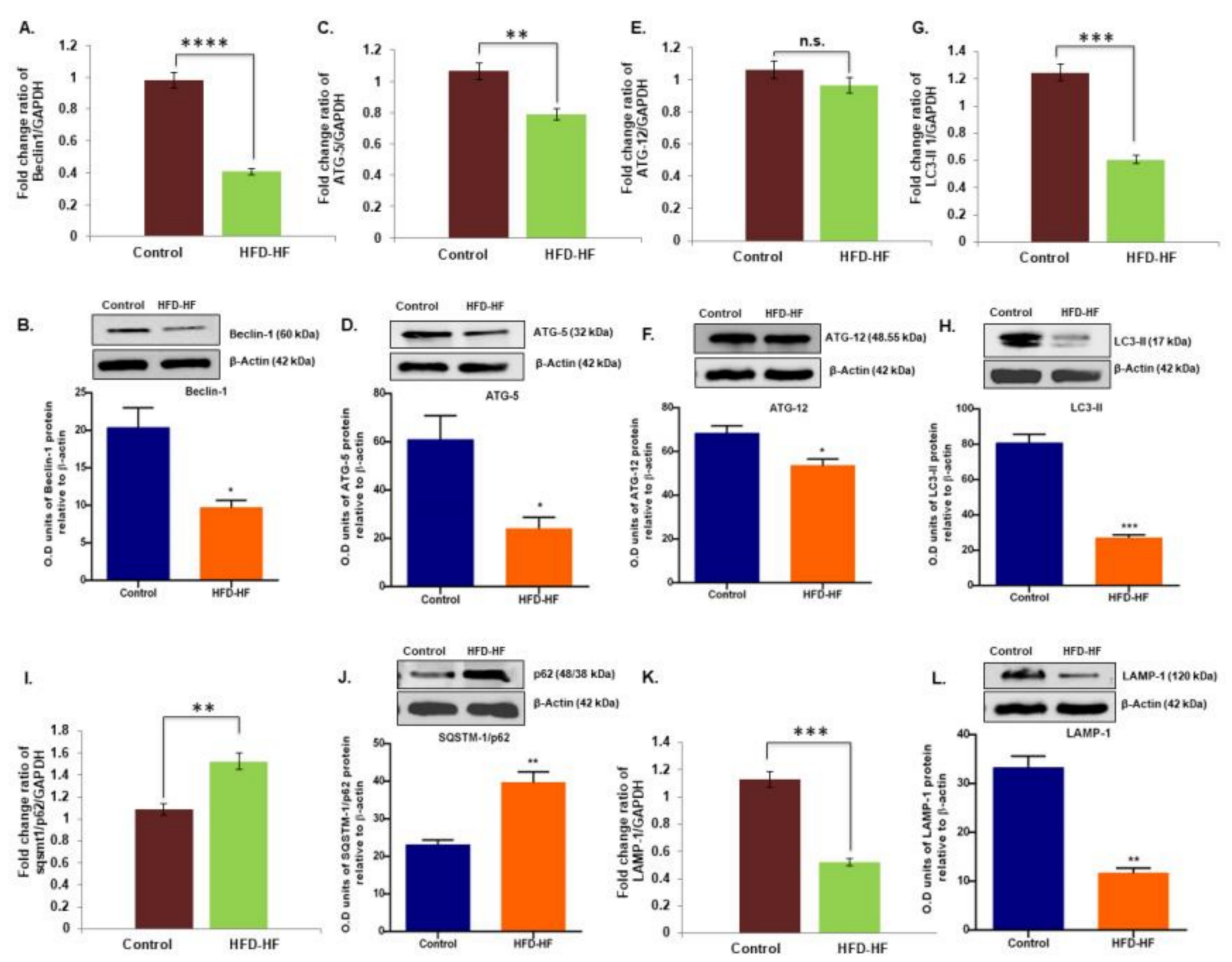
Effects of the HFD-HF diet on the mRNA and protein expression of Beclin-1 (**A**,**B**), ATG5 (**C**,**D**), ATG12 (**E**,**F**), LC3-II (**G**,**H**), SQSTM1/p62 (**I**,**J**), and LAMP1 (**K**,**L**), respectively, in the ovaries of adult rats. Bars with n.s. denote statistically insignificant differences between the respective means, while those with asterisk (*, **, ***, ****) denote statistically significant differences (* *p* ≤ 0.05, ** *p* ≤ 0.01, *** *p* ≤ 0.0001, **** *p* ≤ 0.0001 vs. control group) between the respective means.

The mRNA and protein expression (Figure 8A–L) of the key autophagy regulating genes Beclin1, Atg5, Lc3-II, and Lamp-1 significantly decreased (*p* ≤ 0.05), whereas Sqstm1/p62 expression elevated in the ovary of the HFD-HF diet-fed rats, compared to control. Interestingly, the mRNA expression of the Atg12 gene did not change in the experimental rats, although Western blotting detected a significant decrease (*p* ≤ 0.05) in ATG12 protein (Figure 8E,F).

## 4. Discussion

The current study revealed that an HFD-HF diet interferes with the regulation of GnRHR in the anterior pituitary leading to attenuation of gonadotropins (LH and FSH), which impairs the autophagy-mediated differentiation of ovarian follicles and ovulatory processes. The pubertal onset in females normally occurs on PND 32–PND 38 [28] and is indicated by the appearance of first estrous and the age of vaginal opening, which is gonadotropin-dependent [29,30,31]. In the present study, delayed vaginal opening (PND 55–PND 60) and irregular cyclicity with a short diestrous phase are attributed to decreased LH and FSH levels. Interestingly, the body weight of HFD-HF-fed rats did not show significant alteration, compared to control, albeit various studies reporting weight gain [32,33] and weight loss [34] due to a high-fat diet exposure. Nevertheless, assorted studies have shown altered follicle counts, impaired oocyte quality, the increased accumulation of lipids in cumulus cells and oocytes, and impaired ovulation and fertilization due to high dietary fats without significant changes in the body weight [35,36]. We noticed reduced ovarian quotiety (associated with E2 reduction [37]) and the defective differentiation of ovarian follicles, including maturation and ovulation problems, despite unaltered body weight due to the HFD-HF diet. We assume the discrepancies in the body weight may be due to distinct factors, such as the percentage of fat in the food, different models of rats (Wistar, SD rats) for experiments, etc. The findings suggest that the HFD-HF diet may have aided in reducing reproductive capacity.

The histomorphology of the ovary of HFD-HF-fed rats exhibited reduced ovarian organ index and ovarian area. Furthermore, there was a decreased number of primordial follicles, primary and secondary follicles in the ovary of HFD-HF-fed rats which may be linked to reduced aromatase [31] and E2 level as it facilitates the formation of primary follicles from germ cells nest and their activation from the primordial pool. The revelation of reduced theca interstitial and disintegrated GCs layer in the ovary of the HFD-HF diet, indicative of ovarian dysfunctions, may be attributed to the decrease in gonadotropins [38]. However, the atresia of pre-antral and antral follicles may be associated with reduced androgen and E2, and the status of their respective receptors AR and ER. Abundant AR, present on pre-antral and antral follicles and androgen regulates their number by preventing atresia [39]. Moreover, E2 inhibits pro-apoptotic markers p53 and Bax by preventing apoptosis and subsequently follicular atresia [40,41]. Thus, lower levels of T, E2, AR, ER, and aromatase in the ovary of HFD-HF rats may explain the cause of follicular atresia. The ovary of HFD-HF-fed rats possessed numerous follicular cysts with flattened GCs encompassing follicular fluid without oocytes, indicating hampered ovulation. The decreased number of CLs and lower E2 and P4 levels suggest a cystic transformation of follicles. It is established that ovulation requires the expression of PR [42], which is activated by LH and the secretion of P4. Mice null for PR failed to ovulate even when stimulated with exogenous hormones [43]. In the present study, the decreased number of CLs and the reduction of P4 and PR indicate failed ovulatory processes leading to luteal dysfunctions and thus pregnancy outcomes in the HFD-HF-fed rats.

**Scheme 1:**
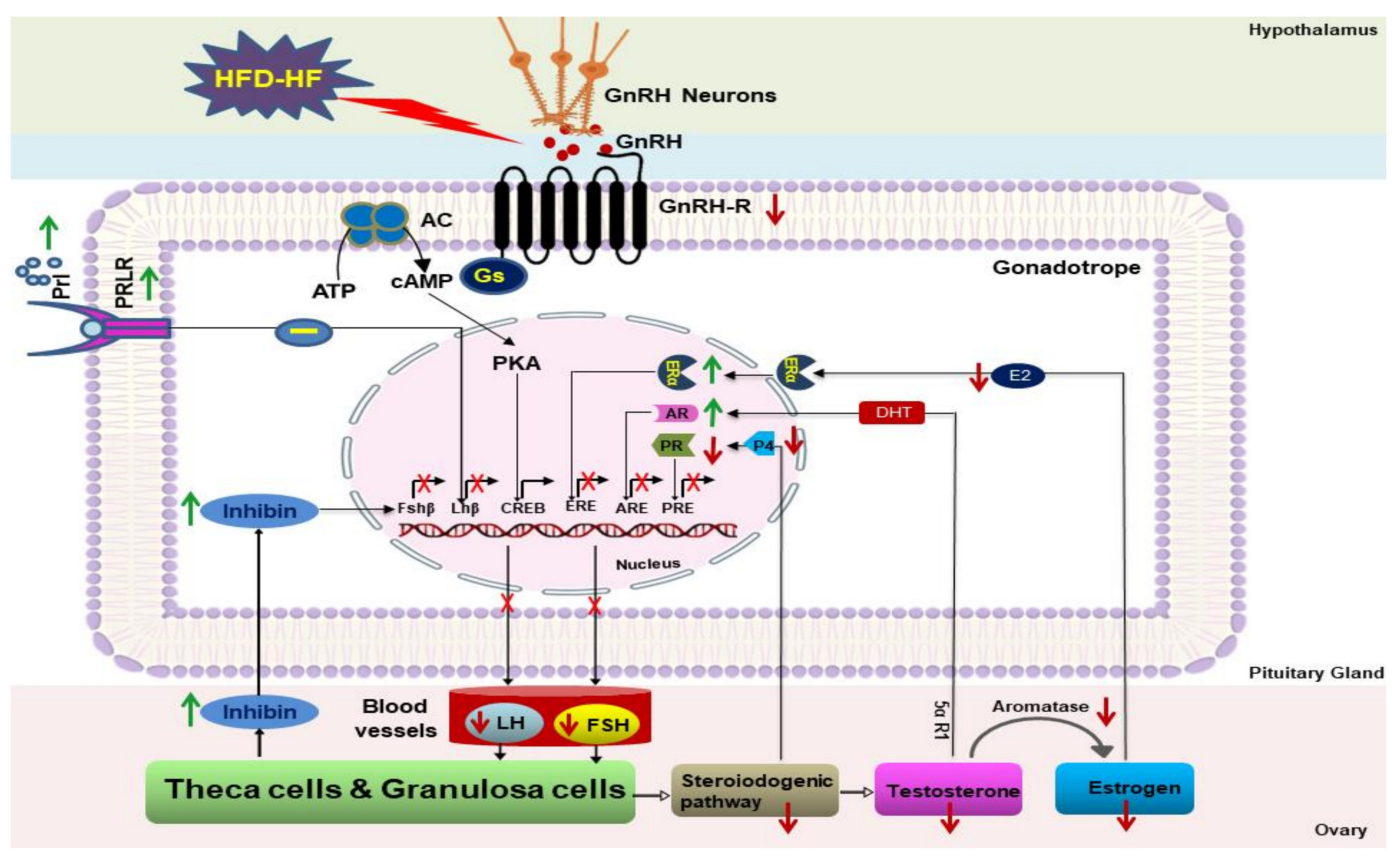
Effects of HFD-HF diet on the pituitary–ovarian axis.

GnRH receptor (GnRHR) on pituitary gonadotropes connects hypothalamic GnRH pulses to ovarian response. GnRH prominently regulates gonadotropin status through its receptor GnRHR on gonadotropes. In the present study, the HFD-HF diet decreased FSH and LH levels (hypogonadotropism), which may be due to the reduced GnRHR. However, steroid hormones through their specific receptors also regulate GnRHR and gonadotropin levels. E2 augments GnRHR numbers [43,44], although the GnRHR promoter has no estradiol-responsive element (ERE) in rats and mice [45,46]. Furthermore, P4 suppresses Gnrhr transcription and reduces pituitary responsiveness [43,47]. While the GnRHR promoter lacks the progesterone response element (PRE) in rats [45,46], it nevertheless responds to P4, and P4 drop results in increased GnRHR [48,49,50,51]. In addition, E2 and P4 regulate the synthesis and secretion of LH and FSH by enhancing the activity of Lhb and Fshb in LβT2 cells [38,52]. In the pituitary, locally aromatized E2 produces the feedback regulation of LH and FSH [53], predominantly mediated through ERα [54]. Thackray et al. (2010) demonstrated an inhibitory effect of P4 on the LHβ subunit promoter in LβT2 cells, although the stimulatory effect of P4 on the basal transcriptional activity of FSHβ and LHβ subunits were not modulated in the presence of E2 [55]. In the current study, the decreased level of E2, P4, PR, and aromatase in the HFD-HF-fed rats suggests that the attenuation of GnRHR, as well as LH and FSH, may not be attributed to these hormones and associated receptors. Furthermore, androgen positively regulates pituitary GnRHR as it contains an androgen response element (ARE) on the proximal promoter [56], and thus it directly regulates GnRHR through the recruitment of AR [57]. Moreover, androgens have a biphasic effect on pituitary gonadotropin output, increasing FSH secretion while decreasing LH secretion. Androgens have a stimulatory effect on FSH [58], suggesting that the decrease of T despite the fact that increased AR may not be involved in the attenuation of GnRHR and reduced secretion of FSH in HFD-HF-fed rats. Inhibin β, a specific inhibitor of FSH synthesis and secretion [58,59], is expressed in the pituitary [55,60,61] as well as in the ovary [62]. A reduced FSH level in the HFD-HF-fed rats may be associated with the elevated inhibin β in the pituitary. Hyperprolactinemia lowers gonadotropin secretion. PRL has a direct inhibitory effect on the gonadotropes, as augmented PRL level impaired GnRHR responses to GnRH and led to reduced gonadotropin (LH) secretion [57]. Increased PRL (an inhibitor of gonadotropin) and decreased E2 discourage GnRH and thus GnRHR [63]. It appears that elevated PRL and its receptor (PRLR) in the gonadotropes are involved in the lowering of GnRHR and LH levels in the HFD-HF-fed female rats (Scheme 1).

**Scheme 2:**
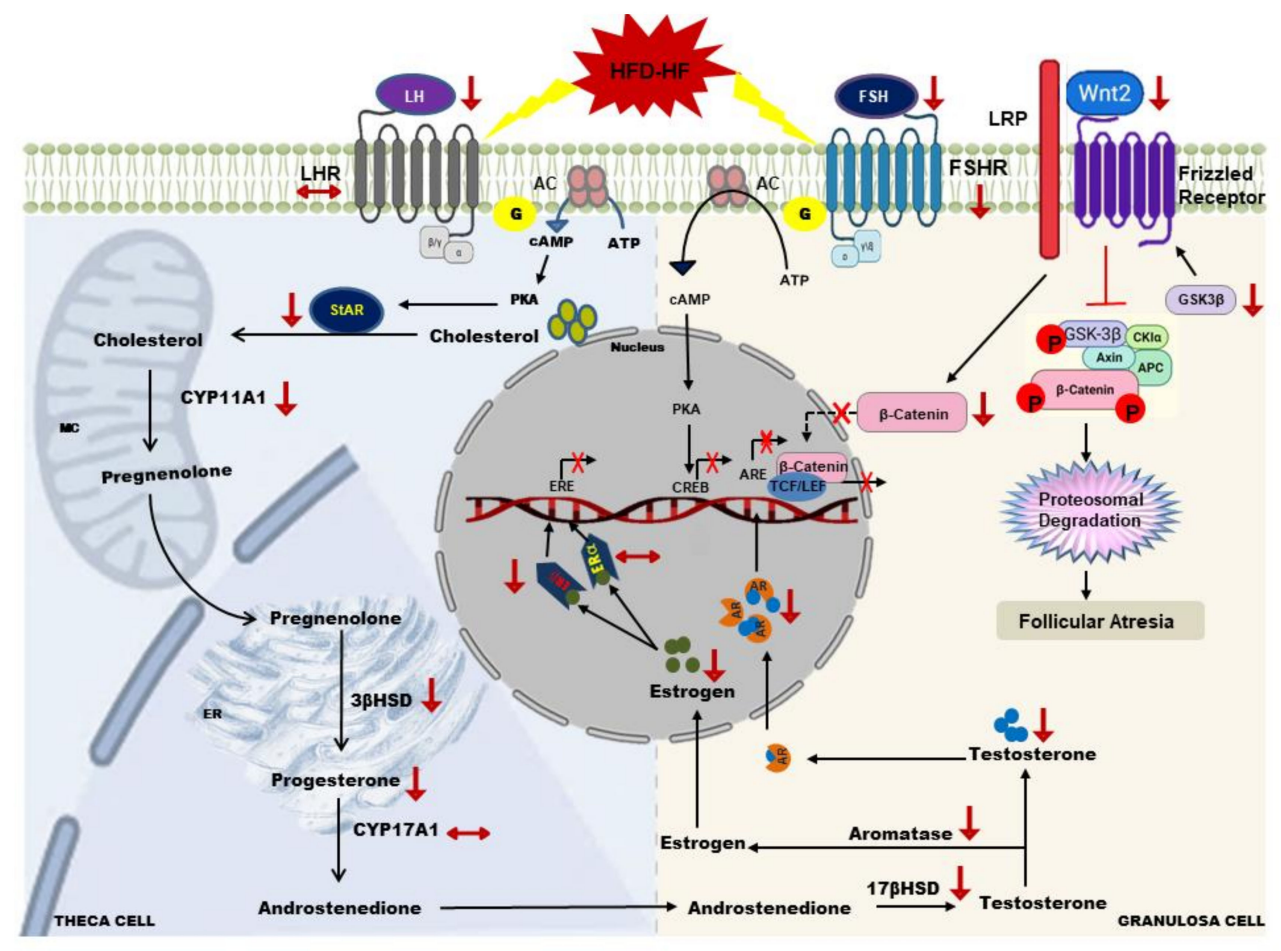
Effects of HFD-HF diet on the ovarian granulosa and theca cells.

Gonadotropins regulate steroidogenesis, follicle development, oocyte maturation, ovulation, and the formation of the corpus luteum [39,64] (Scheme 2). In the steroidogenic pathway, the StAR protein regulates P4 synthesis by enhancing the conversion of cholesterol (the precursor for pregnenolone) into pregnenolone. Our data show that the HFD-HF diet decreased the expression of StAR protein in ovarian cells. This might have resulted in the limited availability of cholesterol inside the mitochondria to be converted to P4 [65,66]. Moreover, an increase in transcriptionally active β-catenin interacts with the StAR gene promoter and boosts StAR mRNA level and P4 synthesis [15], although it requires gonadotropins contribution to maximally impact steroidogenesis. Down the steroidogenic pathway, the HFD-HF diet also decreased CYP11A1 (converts cholesterol to pregnenolone) and 3βHSD (converts pregnenolone to progesterone), which resulted in P4 decline. Thus, in the current study, increased pGSK3β and decreased β-catenin along with lowered LH levels may explain the reduced level of StAR, CYP11A, and 3βHSD that led to the reduction of P4. Interestingly, 17βHSD (converts androstenedione to T) also decreased, which led to a decline in T level. The diminution of the key steroidogenic enzymes and the resultant decline in P4 and T levels suggests that HFD-HF diet-induced hypogonadotropism and a compromised Wnt2/GSK3β/β-catenin pathway disrupt the steroidogenic machinery in the ovary of adult rats.

Gonadotropin receptors, FSHR and LHR, are indispensable for ovarian follicular development. Evidence shows FSHR knockout mice were infertile, displaying small ovaries with the blockage of folliculogenesis before the antral follicle stage, while LHR knockout mice delayed vaginal opening and had small ovaries without follicles at the preovulatory stage and CL [67]. Further, androgen attenuation leads to follicular atresia by enhancing miR125b, which suppresses pro-apoptotic protein and enhances FSHR expression and, thus, FSH-mediated follicles growth and development [68]. In addition, E2 induces gonadotropin, crucial for oocyte maturation and ovulatory induction [64], and interacts predominantly with ERβ and also ERα [69]. The loss of ERβ has been linked to lower E2 and an attenuated preovulatory gonadotropin surge to complete the failure of ovulation [64]. In ERβ KO mice and rats, steroidogenesis and follicle maturation were reduced, which was associated with an attenuated gonadotropin surge [70,71]. In the current study, decreased FSH and its receptor, LHR, T, AR, E2, and ERβ suggest that the HFD-HF diet impairs ovarian functions, especially the development of follicles and their maturation and ovulatory processes.

Wnt signaling (β-catenin and GSK3β) and FSH synergistically promote cell proliferation and E2 biosynthesis [72]. Wnt2, highly expressed in the preantral and antral follicles [73], was demonstrated to be transcriptionally augmented with FSH treatment in GCs [74]. The knockdown of Wnt2 by RNAi inhibited GCs proliferation, whereas its overexpression promoted GCs proliferation and augmented β-catenin [75]. Moreover, β-catenin enhances the FSH-mediated induction of Cyp19a1 in rat GCs [76] as well as proliferation [73]. In addition, the conditional deletion of β-catenin in mouse GCs reduced FSH to promote Cyp19a1 expression subsequently influencing E2 production [77]. In the current study, the decrease of β-catenin along with FSH may have contributed to the reduction of CYP19A1 and E2 levels. Moreover, decreased Wnt2, β-catenin, and augmented pGSK3β revealed compromised Wnt/GSK3β/β-catenin signaling, which may be the cause of arrested antral follicles growth and follicular maturation in the ovarian GCs of adult rats exposed to HFD-HF diet.

**Scheme 3:**
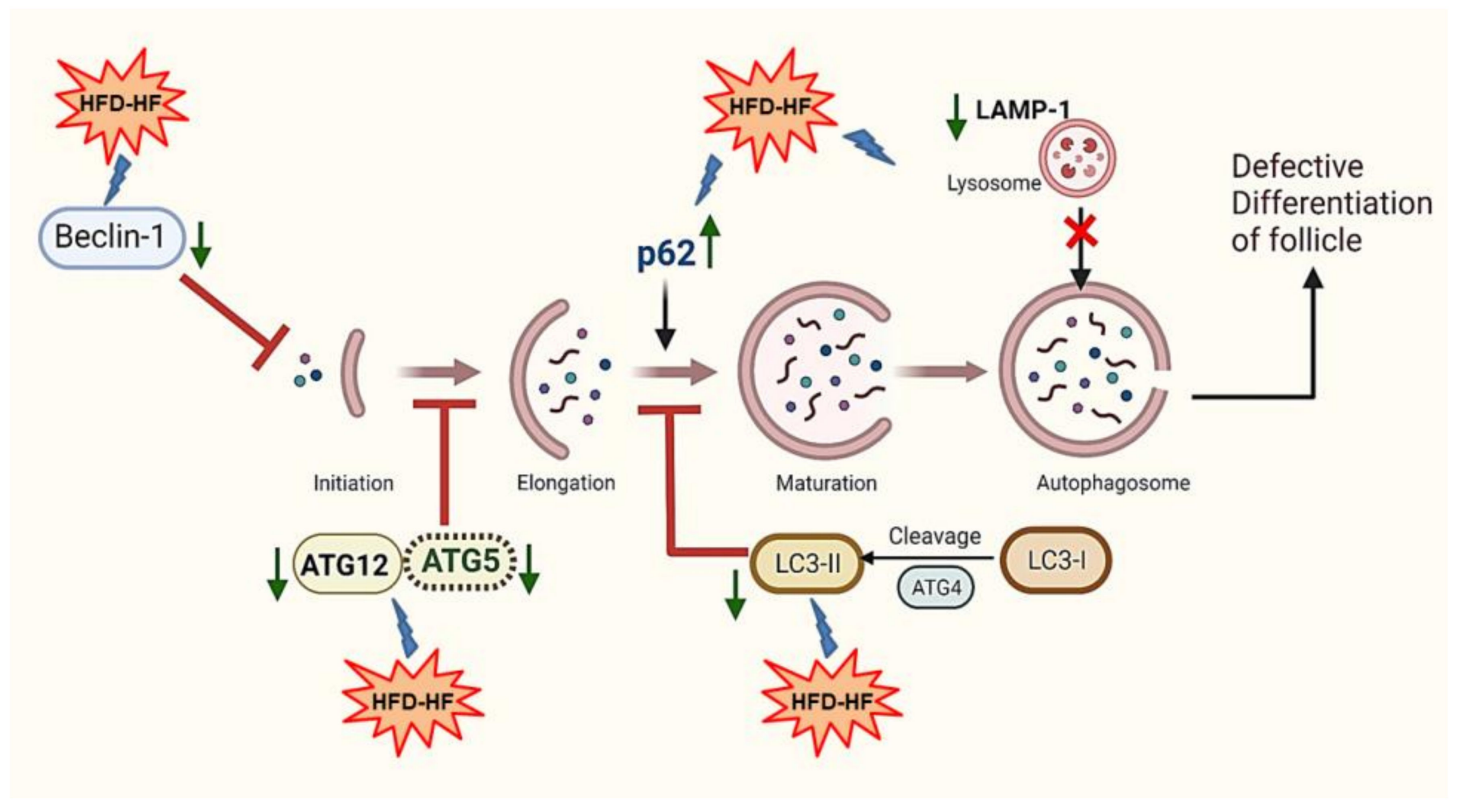
Effects of the HFD-HF diet elicited the inhibition of autophagy, leading to the defective differentiation of ovarian follicles.

Autophagy, a cellular degradation pathway, is essential for the proper development of follicles in the ovary. The components of autophagy machinery consist of the phagophore, autophagosome, and autophagolysosome, which are regulated at each stage by specific marker genes. In a study with a human GC line, the siRNA inhibition of two prominent genes ATG5 and/or Beclin-1 decreased the ratio of LC3II:LC3I expression and elevated the SQSTM1/p62 gene, resulting in the efficient inhibition of autophagy [11]. In the current study, the HFD-HF diet reduced the autophagy biomarkers Beclin-1, ATG5, ATG12, and LC3-II, and elevated the SQSTM1/p62 gene in the ovaries of adult rats, indicating the inhibition of autophagy. Since SQSTM1/p62 is a ubiquitin and MAP1LC3-binding protein that regulates protein aggregate formation in order to be used for autophagic degradation [78], the elevated level of SQSTM1/p62 implies the accumulation of protein aggregates and failure of autophagy at the autophagosome level. In addition, LAMP1, a lysosome marker gene also decreased in the ovary of HFD-HF-fed rats, suggesting that the interaction of autophagosome to lysosome may have hindered and resulted in the inhibition of autophagy. Moreover, a high-fat diet (HFD, 60% kcal in fat) altered membrane lipid composition and downregulated autophagy by reducing autophagosome/lysosome fusion [79] as well as by decreasing the number and the acidity of lysosomes [80] (Scheme 3).

The proliferation, differentiation, and steroidogenic capacity of GCs are crucial for folliculogenesis [81]. Autophagy insufficiency inhibits the differentiation markers of GCs. Shao et al. (2021) demonstrated decreased levels of CYP19A1, FSHR, GATA4, GATA6, SF1, and SP1 genes, and steroidogenic enzymes 3βHSD and StAR in the ATG5 or Beclin-1 knocked down KGN cells [12]. In the current study, the decreased expression of ATG5 and Beclin-1, as well as FSHR and aromatase; steroidogenic proteins StAR and 3βHSD; and E2 and P4 levels occurs, indicating that autophagy inhibition may have been involved in the defective differentiation of the ovarian follicles of adult rats fed with HFD-HF diet. Abnormal follicular differentiation impairs ovarian function and is one of the reasons for human reproductive disorders, such as premature ovarian insufficiency [82,83]. Insufficient autophagy of granulosa-luteal cells in mice resulted in a reduced level of P4, which led to preterm delivery [21]. Moreover, the downregulation of autophagy was also apparent in premature ovarian insufficiency patients, as evidenced by the lower LC3 and higher SQSTM1/p62 gene, which prevented GCs differentiation [11]. Thus, our data ascertain that the inhibition of autophagy may have impaired the follicular differentiation in the ovary of the HFD-HF-fed rats.

Autophagy in GCs is directly linked to ovarian follicular atresia [17]. Autophagy-mediated apoptotic cell death occurs through the accumulation of autophagosomes in ovarian GCs [84] and luteal cells [20] by lowering Bcl-2 expression and subsequently activating caspases [85]. Moreover, evidence shows the co-localization of LC3-II and cleaved caspase-3 in PMSG-primed immature rat ovaries, particularly in antral and atretic follicles [85], further confirming a close relationship between autophagy and apoptosis in rat GCs, as well as its dependence on gonadotropin. In the present study, the accumulation of autophagolysosomes due to the augmentation of SQSTM1/p62 and the diminution of key autophagic biomarkers indicates the involvement of autophagy inhibition, which may have led to apoptosis and resulted in the follicular atresia in the ovary of HFD-HF diet-fed rats. However, we warrant further investigations with apoptotic markers (Bcl2, Bax, Caspases) to substantiate our findings about follicular atresia in GCs.

## 5. Conclusions

The HFD-HF diet alters the status of hormones and their receptor proteins expression and thus adversely affects the feedback mechanism on the pituitary–ovarian axis which controls reproductive functions. The decrease in GnRHR status in the adenohypophysis of rats subjected to the HFD-HF diet leads to hypogonadotropism, which adversely affects ovarian follicular development, maturation, ovulation, and also steroidogenesis. The synergistic effect of the HFD-HF diet inhibits the autophagy mechanism, resulting in the defective differentiation of ovarian follicles. The decrease in ovary weight, hypogonadotropism, hypogonadism, hyperprolactinemia, and decline in ovulatory processes in the ovary of HFD-HF diet rats indicate subfertility/infertility. This study may provide valuable information for the health risk assessment in children considering the vulnerability of development with exposure to the HFD-HF diet.

## Figures and Tables

**Table 1:** Real-time PCR primers.

Gene Name	Forward Sequence	Reverse Sequence	NCBI Accession No.	Amplicon Size, bp
Becn1	5′ CTCGTCAAGGCGTCACTTCT 3′	5′ CCTCCATTCTTTAGGCCCCG 3′	NM_053739	200
LC3-II	5′ CTCCCAAGAAACCTTCGGCT 3′	5′ AAGCCTAACAAGACTGGCCC 3′	NM_199500	215
Atg5	5′ TCCGTGCAAGGATGCAGTT 3′	5′ GCCGTTCAGTTGTGGTCTGA 3′	NM_001399019	185
Atg12	5′ AAGATGGCAGAAGACCCAGAGG 3′	5′ TCCACAGCCCATTTCTTCGTT 3′	NM_001038495.1	233
Lamp1	5′ TTTCCCAATGCCAGCTCCAA 3′	5′ TAGCGTCCCAGAGCACAATG 3′	NM_012857	139
Sqstm1/p62	5′ CTGTGGTGGGAACTCGCTAT 3′	5′ ATCAGAGAGGTGGCCAAAGG 3′	NM_181550	131
Gapdh	5′ GCATCTTCTTGTGCAGTGCC 3′	5′ GATGGTGATGGGTTTCCCGT 3′	NM_017008	262

## Data Availability

We declare that the data supporting the findings of this study are available within the paper. The data are also available from the corresponding author upon request.
